# Safety, Immunogenicity and Protective Activity of a Modified Trivalent Live Attenuated Influenza Vaccine for Combined Protection Against Seasonal Influenza and COVID-19 in Golden Syrian Hamsters

**DOI:** 10.3390/vaccines12121300

**Published:** 2024-11-21

**Authors:** Ekaterina Stepanova, Victoria Matyushenko, Daria Mezhenskaya, Ekaterina Bazhenova, Tatiana Kotomina, Alexandra Rak, Svetlana Donina, Anna Chistiakova, Arina Kostromitina, Vlada Novitskaya, Polina Prokopenko, Kristina Rodionova, Konstantin Sivak, Kirill Kryshen, Valery Makarov, Larisa Rudenko, Irina Isakova-Sivak

**Affiliations:** 1 Institute of Experimental Medicine, Saint Petersburg 197022, Russia; fedorova.iem@gmail.com (E.S.); matyshenko@iemspb.ru (V.M.); kotomina@iemspb.ru (T.K.); rak.ay@iemspb.ru (A.R.); novitskaya.vv@iemspb.ru (V.N.); rudenko.lg@iemspb.ru (L.R.); 2 Smorodintsev Research Institute of Influenza, Saint Petersburg 197376, Russia; konstantin.sivak@influenza.spb.ru; 3 Research-and-Manufacturing Company “Home of Pharmacy”, Saint Petersburg 188663, Russia; kryshen.kl@doclinika.ru (K.K.); makarov.vg@doclinika.ru (V.M.)

**Keywords:** LAIV, influenza virus, SARS-CoV-2, bivalent vaccine, preclinical research, Syrian hamster

## Abstract

Background/Objectives: Influenza viruses and SARS-CoV-2 are currently cocirculating with similar seasonality, and both pathogens are characterized by a high mutational rate which results in reduced vaccine effectiveness and thus requires regular updating of vaccine compositions. Vaccine formulations combining seasonal influenza and SARS-CoV-2 strains can be considered promising and cost-effective tools for protection against both infections. Methods: We used a licensed seasonal trivalent live attenuated influenza vaccine (3×LAIV) as a basis for the development of a modified 3×LAIV/CoV-2 vaccine, where H1N1 and H3N2 LAIV strains encoded an immunogenic cassette enriched with conserved T-cell epitopes of SARS-CoV-2, whereas a B/Victoria lineage LAIV strain was unmodified. The trivalent LAIV/CoV-2 composition was compared to the classical 3×LAIV in the golden Syrian hamster model. Animals were intranasally immunized with the mixtures of the vaccine viruses, twice, with a 3-week interval. Immunogenicity was assessed on day 42 of the study, and the protective effect was established by infecting vaccinated hamsters with either influenza H1N1, H3N2 or B viruses or with SARS-CoV-2 strains of the Wuhan, Delta and Omicron lineages. Results: Both the classical 3×LAIV and 3×LAIV/CoV-2 vaccine compositions induced similar levels of serum antibodies specific to all three influenza strains, which resulted in comparable levels of protection against challenge from either influenza strain. Protection against SARS-CoV-2 challenge was more pronounced in the 3×LAIV/CoV-2-immunized hamsters compared to the classical 3×LAIV group. These data were accompanied by the higher magnitude of virus-specific cellular responses detected by ELISPOT in the modified trivalent LAIV group. Conclusions: The modified trivalent live attenuated influenza vaccine encoding the T-cell epitopes of SARS-CoV-2 can be considered a promising tool for combined protection against seasonal influenza and COVID-19.

## 1. Introduction

Seasonal influenza virus epidemics result in 290–650,000 excess lethal cases per year, according to WHO statistics [1]. To date, annual immunization seems to be the most effective strategy for influenza prevention and decreasing the economic burden of influenza-related medical cases. Influenza viruses of three subtypes cocirculate globally in the human population: H3N2, H1N1 and B (since 2021, only B/Victoria lineage viruses have been in circulation [2]). Antigenic drift of the viruses of all three subtypes requires updating the composition of the vaccines for influenza prevention twice a year, before the start of the epidemic season in the Northern and Southern Hemispheres.

In addition to influenza viruses, SARS-CoV-2 coronaviruses have been circulating worldwide since 2019. These viruses are also under the pressure of population immunity raised due to the natural infections and mass vaccination with Spike-based COVID-19 vaccines. This has led to the emergence of variants antigenically different from previously circulating viruses, and the neutralizing activity of the sera of convalescents and previously immunized people is decreased against new emerging variants, thus limiting the effectiveness of the available vaccines [3]. It is clear that COVID-19 vaccines also need regular updates of vaccine strain composition, as do seasonal influenza vaccines. As annual immunization campaigns have demonstrated efficacy for influenza prevention in different parts of the world, this infrastructure can be adapted for vaccination against both respiratory pathogens. This can be accomplished either by coadministration of the two licensed vaccines [4] or by developing combination vaccines that simultaneously target influenza and SARS-CoV-2 prophylaxis [5].

Licensed influenza and COVID-19 vaccines are generally designed for intramuscular administration, resulting in a systemic immune response with serum antibodies as the primary indicator of protection. However, this is what drives the evolution of the virus and plays a key role in the emergence and spread of drifted viral variants. An alternative strategy aims to induce a local immune response involving effective stimulation of T-cell immunity, which is exploited by live attenuated and vector vaccines when administered intranasally. Particularly, we used a live attenuated influenza vaccine (LAIV) strain as a vector system for the development of a bivalent vaccine for SARS-CoV-2 and influenza prevention. Previously, we developed and evaluated in animal models a panel of H7N9 LAIV-vectored vaccine prototypes expressing various T-cell cassettes of SARS-CoV-2 [6]. The most successful construct was immunogenic and protected golden Syrian hamsters against both infections. Later, the same SARS-CoV-2 T-cell cassette was used to prepare an H3N2 LAIV-based strain that was studied in vitro and in rhesus macaques [7]. In the current study, we used the same strategy to develop a combined trivalent formulation aiming at simultaneous protection against three seasonal influenza viruses and SARS-CoV-2. The trivalent vaccine contains three LAIV strains: H1N1- and H3N2-based LAIV viruses with insertion of the previously developed SARS-CoV-2 T-cell cassette into the NA segment of the influenza virus [6,7], and the unmodified influenza B/Victoria LAIV strain recommended for current influenza vaccines. Here, we present the results of the evaluation of a modified combined recombinant vaccine in a golden Syrian hamster model. We examined the safety and immunogenicity of the vaccine and its protective potential in a challenge study with all three seasonal influenza viruses and the SARS-CoV-2 virus variants Wuhan, Delta and Omicron.

## 2. Materials and Methods

### 2.1. Viruses and Vaccine Formulations

Wild-type influenza virus A/Guangdong-Maonan/SWL1536/2019 (H1N1), clade 6B.1A5/187V/189E, was purchased from the National Institute for Biological Standards and Control (NIBSC, Potters Bar, Hertfordshire, UK). The A/Brisbane/34/2018 (H3N2) subclade 3c.3a (A/Kansas-14/2017-like virus) virus was received from the WHO Collaborating Centre for Influenza, Melbourne, Australia. The B/Austria/1359417/2021 (B/Victoria), V1a.3a.2 clade wild-type virus was provided by the Worldwide Influenza Centre, The Francis Crick Institute (London, UK).

**Figure 1:**
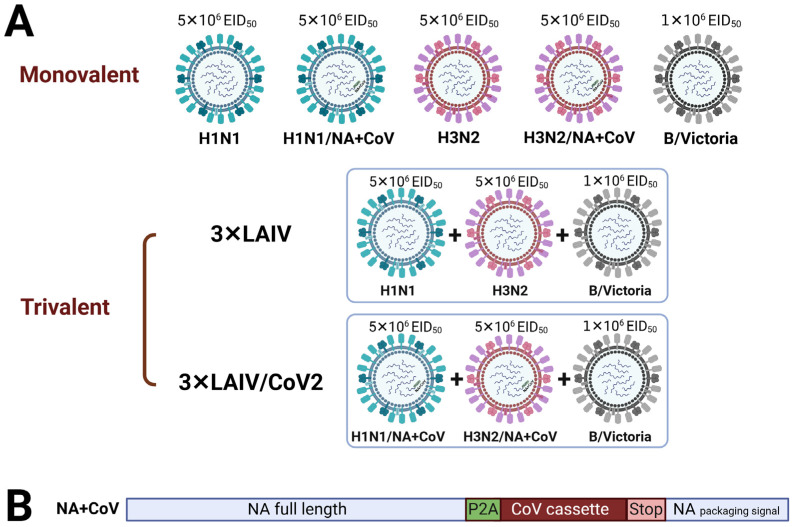
A list of LAIV viruses used in this study. (**A**) Schematic representation of LAIV reassortant viruses either used as monovalent preparations (**upper panel**) or in trivalent compositions (**lower panel**). The dose of each vaccine strain is shown above the virus figure. (**B**) Scheme of the modified influenza A NA gene, where the SARS-CoV-2 T-cell cassette is inserted into the NA open reading frame via the P2A self-cleavage site, which facilitates independent intracellular processing of the influenza NA protein and the inserted cassette [9].

All type A LAIV reassortants were rescued by plasmid-based reverse genetics on a backbone of the A/Leningrad/134/17/57 (H2N2) master donor virus (Len/17 MDV). The standard H1N1 LAIV consisted of two surface genes, HA and NA, from the A/Guangdong-Maonan/SWL1536/2019 (H1N1) strain and the six remaining genes from Len/17 MDV. The classical H3N2 LAIV contained the HA and NA genes from the A/Brisbane/34/2018 (H3N2) virus and the same six remaining genes from Len/17 MDV (Figure 1A). Both chimeric LAIVs, named H1N1/NA+CoV and H3N2/NA+CoV, were modified by the insertion of the SARS-CoV-2 T-cell cassette into the NA open reading frame, as shown in Figure 1B. This selected T-cell cassette comprised the following fragments of the ancestral SARS-CoV-2 strain: N_92–118_ + N_293–370_ + S_948–1016_ (described in more detail in [7]). All the remaining genes of these chimeric viruses were identical to their classical LAIV counterparts (Figure 1A). The viable vaccine viruses were rescued by electroporating Vero cells with a mixture of dual-promoter plasmid DNAs encoding all eight viral segments, as previously described [6].

The type B LAIV reassortant virus was generated by plasmid-based reverse genetics, where plasmids encoding HA and NA genes were derived from the B/Austria/1359417/2021 (B/Victoria lineage) virus and the remaining six plasmids encoded internal and non-structural genes of B/USSR/60/69 MDV [8].

All influenza viruses were propagated in 10–11-day-old embryonated chicken eggs incubated at 33 °C for 72 h. Allantoic fluid was collected, clarified by low-speed centrifugation, aliquoted into single-use vials and stored at −70 °C until use.

SARS-CoV-2 strains hCoV-19/Russia/StPetersburg-3524/2020 (Wuhan D614G variant), GISAID ID EPI_ISL_415710 and hCoV-19/Russia/SPE-RII-32759S/2021 (Delta variant) were received from the WHO National Influenza Centre Russian Federation, Smorodintsev Research Institute of Influenza (Saint-Petersburg, Russia). The Omicron strain was isolated from a human case in St. Petersburg in February 2024. Sanger sequencing of the viral Spike gene identified this isolate as belonging to the JN.1.18 (BA.2.86.1.1.18) Omicron lineage. SARS-CoV-2 variants were grown on Vero E6 cells (African green monkey kidney) obtained from ATCC. The cells were maintained in DMEM supplemented with 10% fetal bovine serum (FBS), antibiotic–antimycotic (AA) and 10 mM HEPES buffer (all obtained from Capricorn Scientific, Ebsdorfergrund, Germany). An 18–12 h cell monolayer was infected with SARS-CoV-2 at a multiplicity of infection (MOI) of 0.001, and the cells were incubated in DMEM with the addition of AA and 2% FBS for 3–5 days until the cells reached full cytopathic effect (CPE). The virus-containing medium was harvested and clarified by centrifugation. Viruses were aliquoted in single-use vials and stored at −70 °C. All experiments with live SARS-CoV-2-containing material were performed in a BSL-3 facility by certified personnel.

### 2.2. Titration of Influenza Viruses and SARS-CoV-2

Influenza virus titers were determined by inoculating 10–12-day-old eggs with ten-fold serial dilutions, according to the standard protocol [10]. Infected eggs were incubated at 33 °C or 38 °C for 3 days or at 26 °C for six days to study the phenotypic properties of the vaccine viruses. Viral titers were calculated based on the number of influenza-positive eggs in each dilution using the Reed and Muench method [11] and were expressed in lgEID_50_/mL (50% egg-infective doses).

Titers of SARS-CoV-2 viruses were assessed in Vero E6 cells using two different assays. In the first assay, the 50% tissue culture infectious dose (TCID_50_) was determined by inoculating Vero E6 cells grown on 96-well plates with serial 10-fold dilutions of each coronavirus. Infected cells were incubated for 5 days, and then virus-positive cells were identified by CPE visible in an optical microscope. Virus titers were calculated using the Reed and Muench method and expressed in lgTCID_50_/mL.

The assessment of SARS-CoV-2 titers by fluorescent focus unit (FFU) assay was performed as described previously [12]. Briefly, Vero E6 monolayers were inoculated with SARS-CoV-2 10-fold dilutions in a 96-well plate format. After 11–19 h incubation, the media were removed and the cells were fixed with 2% formaldehyde. After washing, permeabilization with 0.1% Triton X-100 and blocking non-specific binding with 5% skim milk, the primary anti-N biotinylated monoclonal antibody NCL5 [12] was added to the wells, followed by the addition of EGFP-streptavidin fusion protein. Following incubation and washing procedures, the plates were dried and analyzed using the AID vSpot Spectrum (Advanced Imaging Devices GmbH, Strassberg, Germany). Fluorescent spots were counted in wells with 100–200 spots; virus titers were calculated according to the dilution factor and expressed as lgFFU/mL.

### 2.3. Hamster Study Design

The design of the animal experiments was developed according to the 3R principles and Directive 2010/63/EU [13]. The experimental design was approved by the ethics committee of the research-and-manufacturing company “Home of Pharmacy” (protocol no. 1.20/23, dated 29 May 2023).

The golden Syrian hamsters (*Mesocricetus auratus*), females, nulliparous and non-pregnant, were bred in a nursery in the Home of Pharmacy in the 5th–6th generation, screened for absence of specific pathogens and randomized. Before the experiment, the animals were divided into groups (randomization and stratification by body weight), cocaged in groups (4 per cage) and quarantined; the absence of specific antibodies to influenza and SARS-CoV-2 viruses was assessed by ELISA against influenza A NP protein, influenza B NP protein and SARS-CoV-2 N protein. All recombinant proteins were expressed in E.coli cells as previously described [14].

Animals were immunized intranasally either with one LAIV virus or with mixtures of three classical LAIV strains (3×LAIV) or modified trivalent LAIV (3×LAIV/CoV2) at doses shown in Figure 1A: 5 × 10^6^ for type A LAIVs and 1 × 10^6^ for type B LAIVs. Doses were selected based on previously obtained data on the immunogenicity of each component of trivalent LAIVs in clinical trials, where interference between vaccine strains was observed, with type B LAIVs being consistently more immunogenic than the A/H1N1 and A/H3N2 LAIVs [15,16]. All vaccines were administered twice, with a 3-week interval, in a volume of 50 μL, without anesthesia.

**Figure 2:**
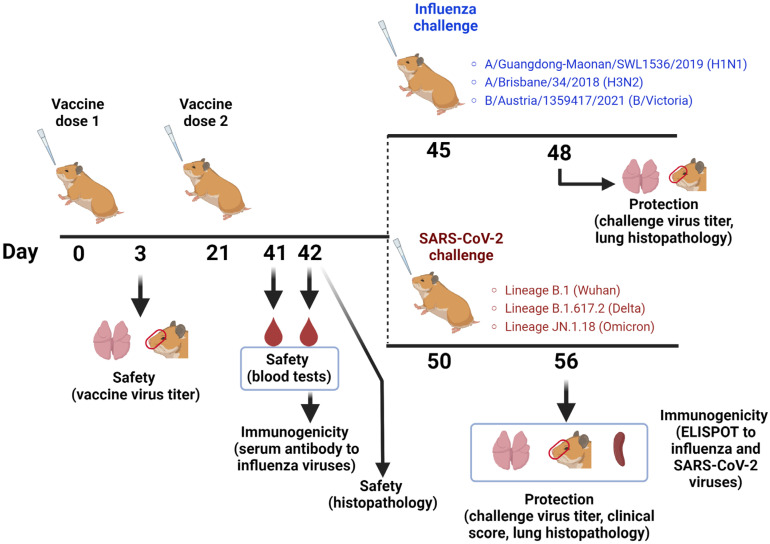
The scheme of the experiment for assessment of the safety, immunogenicity and protective potential of the studied monovalent and trivalent LAIV candidates in Syrian hamsters. Doses of vaccine viruses administered alone or in trivalent compositions are shown on Figure 1A. D—days of the experiment. Red circles denote the extracted nasal turbinate tissues.

At day 3 of the experiment, four animals from each vaccine group were euthanized and respiratory tissues (nasal turbinates, lungs and trachea) were collected for the assessment of viral replicative properties (Figure 2). Organs were stored frozen at −70 °C until viral titration. For this, 1 mL of cold PBS supplemented with 2 × AA was added to each organ, and tissue homogenates were prepared using a small bead mill, the Qiagen TissueLyser LT. Clarified by low-speed centrifugation, the supernatants were used to inoculate eggs, and viral titers were determined as described above.

Serum samples were collected on day 42 of the study (three weeks after the second dose) to assess influenza antibody responses and for blood tests as a measure of vaccine safety.

On day 45 of the study, four animals from each group were infected with one of the three wild-type influenza viruses: A/Guangdong-Maonan/SWL1536/2019 (H1N1), A/Brisbane/34/2018 (H3N2) or B/Austria/1359417/2021, giving a total of 12 animals for each study group. The viruses were administered intranasally at a dose of 10^6^ EID_50_ per animal, in a volume of 100 µL. On day 3 post-infection, the hamsters were euthanized and lungs and nasal turbinates were collected to determine viral loads as described above, with the exception that viral titers were adjusted to the weight of extracted organs and expressed as lgEID_50_/gram tissue. In addition, lung tissues were subjected to histopathological analyses as described below.

Due to the limited capacity of the BSL-3 laboratory, challenge with SARS-CoV-2 variants was performed for three study groups only: the 3×LAIV, 3×LAIV/CoV2 and mock-immunized animals. On day 50 of the study, fifteen animals from each group were infected i.n. with one of three SARS-CoV-2 strains (Wuhan, Delta or Omicron; *n* = 5 each) at a dose of 10^5^ TCID_50_, in a volume of 100 µL. Clinical signs of disease were monitored for six days post-infection, and respiratory tissues were collected on day 6 post-infection for measuring the SARS-CoV-2 viral load in tissue homogenates using the FFU assay. Additionally, half of each lung was transferred to 10% formaldehyde for histopathological studies.

### 2.4. Safety Assessment

The safety of the monovalent and trivalent LAIV compositions was assessed by monitoring body weight and clinical signs of disease during the vaccination period. Additionally, six animals from each group were euthanized on day 42 to collect various organs and tissues for pathomorphological and histologic evaluation, including the assessment of local irritant effects (Figure 2). Blood samples collected at this time point were used for clinical and biochemical blood tests.

#### 2.4.1. Animal Health Status

The hamsters were weighed weekly on days 0, 7, 14, 21, 28, 35 and 42, immediately before euthanasia, to calculate the percentage of organ weight to body weight, using electronic scales, the AJ-1200CE (Vibra, ShinkoDenshi, Japan). Clinical observation of animals was carried out daily, once a day, at the same time from day 0 to day 42 of the experiment. On the days of administration of the investigated objects, clinical observation of animals was carried out within 1–2 h after administration. The following parameters were recorded: (1) animal behavior in the cage: normal/oppression (slight, moderate, significant/agitation (moderate, significant)/aggression/auto-aggression; (2) body position in space, coordination: normal/change of posture/lameness/coordination disorder; and (3) coat condition: normal/soiling/ruffling/alopecia/other. Since during the whole experiment general clinical observation did not reveal clinical signs that indicated a deviation from the normal condition of the animals, a detailed examination was not carried out.

#### 2.4.2. Blood Tests

On day 41 of the experiment, blood samples were collected from the gingival vein in volumes of 200 µL (for hematologic analysis) and 10–20 µL (for biochemical analysis of glucose) from six animals to be euthanized on day 42 of the experiment. Blood was collected in tubes with K3-EDTA (Impromini, China). Vital blood sampling for tests was performed, with the volume not exceeding 10% of the circulating blood volume. During euthanasia on day 42, blood samples were collected from animals by exsanguination of the heart cavities for analysis of biochemical parameters (except glucose). For this, blood was collected in Lind-Vac 3.5 mL tubes (OÜ InterVacTechnology, Estonia). To obtain serum, blood was centrifuged for 15 min at 3000 rpm, followed by serum transfer into new clean tubes.

For clinical blood tests, the following parameters were determined in whole blood samples using a hematology analyzer, the Mythic 18 Vet (Orphee, Switzerland): number of red blood cells (RBC, 10^12^/L), hemoglobin level (HGB, g/L), mean hemoglobin content in erythrocytes (MCH, pg), mean hemoglobin concentration in erythrocytes (MCHC, g/L), hematocrit (HCT, %), platelet count (PLT, 10^9^/L), mean platelet volume (MPV, fl), white blood cell count (WBC, 10^9^/L), thrombocrit (PCT, %), mean red blood cell volume (MCV, fl), lymphocytes (LYM, %), monocytes (MON, %) and granulocytes (GRA, %).

Glucose levels were analyzed using a One Touch Select^®^ Plus glucometer (Lifescan Europe, Switzerland) and test strips corresponding to the device. For evaluation of other biochemical parameters, serum samples were loaded into the Random Access “A-25” analyzer (BioSystems, Spain) to determine the following parameters: alanine transaminase (ALT, U/L), aspartate aminotransferase (AST, U/L), urea (blood urea nitrogen (BUN), mmol/L), total protein (TP, g/L), creatinine (CREA, µmol/L), total cholesterol (CHOL, mmol/L), albumin (ALB, g/L), globulin (GLOB, g/L), triglycerides (TG, mmol/L) and total bilirubin (BIL-T, µmol/L).

#### 2.4.3. Histopathology

In addition to serum collection, on day 42 of the study, various tissues were harvested for pathomorphological and histopathological evaluation (*n* = 6 per group). The pathomorphological examination included necropsy and macroscopic analysis. The necropsy procedure was performed with detailed examination and description of the external condition of the body, skin, hair coat, external passages, place of administration of the investigated objects (nasal passages), visible mucous membranes, and the thoracic, abdominal and pelvic cavities with the organs in them. Immediately after routine euthanasia and necropsy, internal organs were extracted to register their weight and subsequent transfer for histologic examination. The organs to be weighed included the heart, lungs with trachea, thymus, liver, spleen, kidneys, adrenal glands, brain and ovaries. The organs subjected to histopathology evaluation included the heart, trachea, lungs with bronchi, nasal turbinates, larynx, submandibular lymph nodes, thymus, esophagus, stomach, small intestine, large intestine, pancreas, liver, spleen, kidneys, bladder, adrenal glands, ovaries, cephalad and brain. For histological examination of tissues and organs, the material was fixed in 10% neutral formalin solution for 24 h, after which it was poured into paraffin according to the generally accepted technique. For histologic study, nasal passages (nasal turbinates) were decalcified for 14 days. The prepared sections were stained with hematoxylin and eosin, and then these histological preparations were analyzed using an Accu-Scope 3000 SERIES light-optical microscope (ACCU-SCOPE Inc., Commack, NY, USA) at magnifications of 40, 100 and 400. Microphotography was performed using a TOUP-CAM UCMOS05100KPA digital camera (Hangzhou, China) and ToupView 3.7.7892 software (Hangzhou, China).

### 2.5. Immunogenicity Assessment

Anti-influenza immune responses were measured by enzyme-linked immunosorbent assay (ELISA) using the sucrose gradient-purified whole influenza viruses as coating antigens. Specifically, unmodified H1N1 LAIV, H3N2 LAIV and B LAIV viruses were used in this assay. High-sorbent 96-well plates (Corning, NY, USA) were coated with the virus antigens at a dose of 16 hemagglutinating units (HAU) per well overnight at +4 °C. After washing with PBS supplemented with 0.05% Tween 20 (PBST) and blocking non-specific binding with 0.5% bovine serum albumin (BSA) solution, 2-fold dilutions of hamster sera were added to the wells, starting from a 1:10 dilution. After 30 min incubation at 37 °C and extensive washing with PBST, antigen-bound IgG antibodies were detected using horseradish peroxidase-conjugated anti-hamster IgG secondary antibodies (BioRad, Hercules, CA, USA) diluted 1:3000. Following 30 min incubation and a washing procedure, the plates were developed by the addition of 1-Step Ultra TMB-ELISA Substrate Solution (Thermofisher Scientific, Waltham, MA, USA). The reaction was stopped 15 min later by adding 1M H_2_SO_4_ solution, after which the optical density of the solution at 450 nm (OD_450_) was read using an xMark spectrophotometer (BioRad, Hercules, CA, USA). The maximum dilution of the sample at which the OD_450_ value was two times the average OD_450_ of the control wells (antigen without serum addition) was assumed as the serum IgG titer.

Cell-mediated immune responses to influenza viruses and to the SARS-CoV-2 antigens were measured in hamster splenocytes collected six days after SARS-CoV-2 challenge with the Wuhan and Delta variants (day 56 of the study) using the IFN-γ ELISPOT Plus kit (Mabtech, Hamburg, Germany) according to the manufacturer’s protocol. Briefly, isolated splenocytes were filtered through 70 µm cell strainers (Wuxi NEST Biotechnology Co., Ltd., Wuxi, China), and 250,000 cells were added to a sterile U-bottom 96-well plate in 100 µL of CR-10 medium (RPMI supplemented with 10% FBS, 5 mM HEPES, 1× antibiotic–antimycotic and 50 μM β-mercaptoethanol). The ELISPOT plate pre-coated with monoclonal antibodies to hamster IFNγ was washed 4 times with sterile PBS (200 μL/well), then incubated with CR-10 medium for 30 min at room temperature. At the same time, 100 µL of one of the stimuli was added to each well containing splenocytes: (i) sucrose gradient-purified H1N1 LAIV virus; (ii) sucrose gradient-purified H3N2 LAIV virus; (iii) sucrose gradient-purified B LAIV virus; (iv) sucrose gradient-purified SARS-CoV-2 (homologous variant for each challenge); (v) PepTivator^®^ SARS-CoV-2 Prot_S + Prot_N, 30 pmol per peptide (Miltenyi Biotec, Bergisch Gladbach, Germany). Influenza viruses were used at a multiplicity of infection (MOI) of 1–3, whereas SARS-CoV-2 coronaviruses were used at lower doses of MOI = 0.1 and MOI = 0.01. The cells + stimuli mixtures were added to the pre-washed ELISPOT plate, followed by an 18 h incubation at 37 °C, 5% CO_2_. Staining was performed using detection antibodies and the prepared substrate solution according to the manufacturer’s protocol. The development of staining was stopped by abundant rinsing in tap water, after which the plate was left to dry overnight. Stain counting was performed in an AID vSpot Spectrum reader (Advanced Imaging Devices GmbH, Strassberg, Germany). The number of spots from control wells (splenocytes without stimulus) was subtracted from the experimental wells to obtain the final number of spots per 250,000 cells.

### 2.6. Protection Against Influenza Viruses

Protection against influenza virus challenge was assessed by virological and histopathological endpoints. As stated above, respiratory tissues were collected from influenza virus-infected hamsters on day 4 post-challenge (*n* = 4). Viral titers in tissue homogenates were determined by titration in eggs and compared to the control, mock-immunized animals. For histopathological evaluation, half of each lung was subjected to histological processing as described above for safety analyses. To assess microscopic lung injury, a scoring system was applied as described by Osterrieder N. et al. [17]. The damage scoring system included the analysis of three features: (1) lung inflammation, including severity of (I) interstitial pneumonia, (II) bronchitis, (III) bronchial and alveolar epithelial necrosis and (IV) type II alveolar epithelial cell hyperplasia; (2) cellular infiltration with (I) neutrophils, (II) macrophages and (III) lymphocytes, as well as the presence of (IV) perivascular lymphocytic accumulations; (3) pulmonary edema, including (I) alveolar edema and (II) perivascular edema. The following severity scale was applied for each parameter: 0—absent, 1—minimal, 2—mild, 3—moderate and 4—severe.

### 2.7. Protection Against SARS-CoV-2 Infection

Since SARS-CoV-2 infection induces clear clinical signs of disease, protection against this infection included clinical observations, along with the assessment of viral titers in respiratory tissues and histopathological evaluations of lung lesions. The clinical manifestation of the disease was judged by the appearance and behavior of the animals. In particular, coat condition was evaluated as normal (0 points) or as lack of grooming (1 point). Behavioral assessment included interaction with other animals (0, normal; 1, reduced), food consumption (0, normal; 1, reduced), open-space behavior (0, active; 1, reduced) and pick-up reaction (0, normal; 1, reduced).

SARS-CoV-2 titers were measured by titration of lung and nasal turbinate tissues in Vero E6 cells. Homogenization was performed with a small bead mill, the Qiagen TissueLyser LT, and 10^−1^ to 10^−3^ dilutions of clarified supernatants were used to infect cell monolayers grown on 96-well plates. After 11–19 h of incubation, virus-infected cells were visualized using the FFU assay, as described in Section 2.2.

Histopathological evaluation of lung tissues of SARS-CoV-2-challenged hamsters was performed as described earlier [6]. Briefly, formalin-fixed tissues were processed using a Histo-Tek VP1 histoprocessor (Sakura Finetek, Tokyo, Japan), followed by sample embedding in paraffin blocks with subsequent preparation of 3 μm sections and their staining with a hematoxylin-and-eosin solution. Microscopic examination was performed using a LEICA DM1000 light microscope (Leica Microsystems, Wetzlar, Germany). Images were made and analyzed using the ADF Image Capture 4.17 software package. Scoring of the histopathological changes was carried out as described in Table S1.

### 2.8. Statistical Analysis

Intergroup differences were analyzed by parametric or non-parametric methods, depending on the type of distribution. Analysis of variance (ANOVA) was used to analyze the data that followed a normal distribution. For multiple comparisons, Tukey’s post hoc analysis was used. For data that did not conform to the law of normal distribution, the Kruskal–Wallis test was applied. The results were analyzed using GraphPad Prism 7.0 software. The differences were considered significant at *p* < 0.05.

## 3. Results

### 3.1. Characterization of Vaccine Strains Included in the Trivalent LAIV and LAIV/CoV2 Vaccines

The replication of the vaccine strains was evaluated in eggs and MDCK cells, as well as in the respiratory organs of hamsters after intranasal inoculation of 10^6^ EID_50_ of each strain (Table 1). Virus titers in eggs were measured at optimum (33 °C), low (26 °C) and elevated (40 °C) temperatures to assess the virus phenotype. All strains studied replicated well at 26 °C; titers of modified strains were slightly lower than those of non-modified strains. In the MDCK cell culture, all five viruses did not differ in growth characteristics. Virus titers in nasal turbinates of hamsters on day 3 after infection were 3–4 lgEID_50_/g, which confirms the effective replication of the viruses, both modified and non-modified, in the respiratory tract of the Syrian hamsters. Titers in lungs were significantly lower than in the URT, which is a standard safety marker of cold-adapted attenuated influenza strains.

### 3.2. Safety of the 3×LAIV/CoV2 Vaccine in a Syrian Hamster Model

The safety of the new modified trivalent 3×LAIV/CoV2 vaccine was assessed by a number of clinical and laboratory parameters and compared to the monovalent LAIV variants (both classical and chimeric LAIV strains), as well as the standard trivalent 3×LAIV.

#### 3.2.1. Clinical Observations

**Figure 3:**
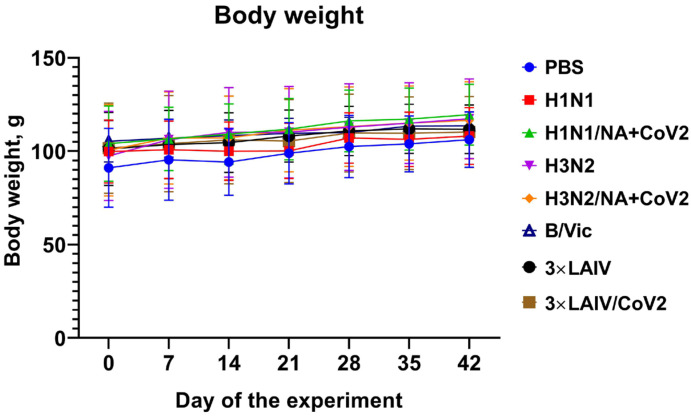
Safety assessment of the modified trivalent 3×LAIV/CoV2 vaccine in Syrian hamsters. Animals were immunized twice with the indicated vaccine variant on days 0 and 21, and body weight was evaluated during the immunization phase, until day 42 of the experiment.

During the experiment, no deaths of animals were recorded, and the hamsters from all study groups were in satisfactory condition: the appearance and behavior of the animals that received the monovalent or trivalent vaccine candidates did not differ from those of the animals in the control group. Respectively, in all animals in each test group the scores for clinical signs of the disease were equal to zero. The body weights of the animals before the beginning of the experiment did not statistically significantly differ between the groups. Administration of any vaccine variant did not affect the body weights of the animals after either the first or the second dose (Figure 3).

Thus, intranasal administration of the studied vaccines at the indicated doses had no negative effect on the general condition of the experimental animals during immunization. In addition, there was no effect of the tested objects in the dose under study on the dynamics of the body weight of the hamsters during this period. Clinically significant differences between the tested objects and the corresponding controls were not observed.

#### 3.2.2. Blood Tests

The results of the clinical blood test on days 41–42 of the study are presented in Table S2. One-way ANOVA revealed significant differences between the study groups in such parameters as WBC, HGB, RBC, HCT, PLT, MCH, MCHC and PCT (*p* < 0.05). Subsequent analysis using Tukey’s criterion revealed a statistically significant decrease in the WBC indicator in the group that received 3×LAIV compared to the control group. In addition, PLT counts were significantly decreased in the groups that received H1N1 LAIV, H3N2/NA+CoV2 LAIV, B LAIV and 3×LAIV/CoV2 compared to the PBS group (Table S2). A decrease in PCT was also observed in the groups that received H1N1 LAIV, B LAIV and 3×LAIV/CoV2 relative to the control animals. Thrombocrit (PCT) is the fraction (%) of whole blood volume occupied by platelets, meaning the two parameters are directly related to each other [18]. Finally, an increase in the MCHC was observed in the H3N2 LAIV group compared to the control animals. In the group with detected changes, the values did not differ from the laboratory norm by more than 10% (Table S2). Importantly, for most of the laboratory parameters with identified differences between groups, only one of six animals had values below the laboratory norm; therefore, these differences were considered as instances of individual variability of indices. For the rest of the indicators, where the influence of the group factor was detected, Tukey’s analysis did not reveal statistically significant differences from the control group (*p* > 0.05).

For biochemical analysis, one-way ANOVA revealed significant differences among groups in the values of ALB, CHOL, TG, BUN and BIL-T (*p* < 0.05; Table S3). Subsequent analysis using Tukey’s test revealed an increase in ALB and BUN values in the animals that received B LAIV and an increase in TG in the groups that received H3N2 LAIV, B LAIV, 3×LAIV and 3×LAIV/CoV2 compared to the control group. The detected changes in ALB and BUN did not differ from the intra-laboratory norms by more than 10%; therefore, the differences can be considered clinically insignificant. Elevated triglyceride levels in conjunction with other lipid parameters such as total cholesterol (CHOL) are considered a marker of cardiovascular risk as well as risk for acute pancreatitis [19]. However, taking into account the results of other blood biochemical parameters, as well as the results of histologic examination (no effect on the heart and pancreas), we can conclude that the detected change in triglycerides was not clinically significant and was not associated with exposure to the vaccine candidates.

Overall, the studied vaccine variants had no clinically significant effect on the indices of the general and biochemical blood analyses of hamsters after two-dose immunization. No clinically significant differences between the vaccine variants at equivalent doses were observed.

#### 3.2.3. Pathology and Histopathology

Various tissues and organs were collected from six hamsters from each group three weeks after the second immunization to determine whether pathological changes occurred after administration of the study vaccines. The results of estimation of mass factors of internal organs are presented in Table S4. One-way ANOVA revealed significantly lower mass coefficients of the liver in groups H1N1 LAIV and H3N2/NA+CoV2 LAIV and an increase in the mass coefficient of the adrenal glands in group H3N2/NA+CoV2 LAIV relative to the control group. According to the literature, liver weights ranged from 3.42 ± 0.47 g to 4.94 ± 0.26 g (M ± SEM) in studies conducted on Syrian hamsters [20,21,22]. The absolute liver values in the group that received H1N1 LAIV were 3.81 ± 0.27 g and those in the group that received H3N2/NA+CoV2 LAIV were 3.6 ± 0.35 g, which is consistent with data from the literature. Similarly, data from the literature suggest that the main pathology of the adrenal glands is neoplasia, which can lead to increased organ mass [23,24]. Since pathomorphological study did not reveal a pathological effect of either studied LAIV on the liver and adrenal glands, the detected changes in these groups can be considered as instances of individual variability of parameters and are clinically insignificant.

Thus, the assessed vaccines at the indicated doses had no clinically significant effect on the mass organ ratios of the hamsters after two-dose immunization. No clinically significant differences were observed between the study vaccines and the corresponding control groups.

Pathoanatomical and microscopic changes during planned necropsy were found in some animals in different experimental groups (Table S5). Thus, cystic formations on the ovaries were found in one animal from the B LAIV and 3×LAIV/CoV2 groups. Of note, this is a spontaneous background pathology characteristic of Syrian hamsters [25,26]. One hamster in the 3×LAIV/CoV2 group was also found to have polycystic liver, but these changes can often be observed in hamsters and do not relate to the administered vaccine [27]. Minor changes in the kidneys (a small group of dilated convoluted tubules, epithelium flattened, homogeneous eosinophilic infiltration in the lumen and weak inflammation at the periphery of the focus) were detected in one animal from the H1N1/NA+CoV2 and H3 LAIV groups, as well as in two animals out of six in the B LAIV group. These changes can also be attributed to spontaneous pathology characteristic of hamsters [25].

More changes were registered in the lungs of the experimental animals, including single-spot hemorrhages in different lung lobes in individual hamsters from the PBS, H3N2 LAIV, H3N2/NA+CoV2 LAIV and 3×LAIV/CoV2 groups. In one to three animals from each study group, including the mock-immunized hamsters, multiple areas of lung tissue recession and emphysema (distelectasis) were present, mainly in peripheral areas, combined with moderate hemorrhages (Table S5). Since pulmonary hemorrhages and dystelectasis were detected in all the experimental groups, including the group that received PBS, these pathological changes are probably not related to the administration of the test objects. One of the possible reasons for the appearance of pulmonary hemorrhages may be the dying process of animals via the chosen method of euthanasia. According to data in the literature, multiple hemorrhages and moderately pronounced emphysema may be observed in the lungs when animals are euthanized by overdosing with a combination of Zoletil and Xyla [28]. There was also one animal in the 3×LAIV group that was diagnosed with lobular pneumonia progressing to the stage of pulmonary consolidation, but this pathology can arise spontaneously [29] and was not related to the administration of the vaccine.

Importantly, no pathological changes were found at the site of vaccine administration (nasal turbinates) after two-dose immunization.

Overall, the detected changes in the internal organs of hamsters belong to spontaneous pathologies characteristic of this animal species; therefore, they cannot be considered a result of the action of the tested vaccines and are clinically insignificant.

### 3.3. Influenza-Specific Antibody Immune Responses

**Figure 4:**
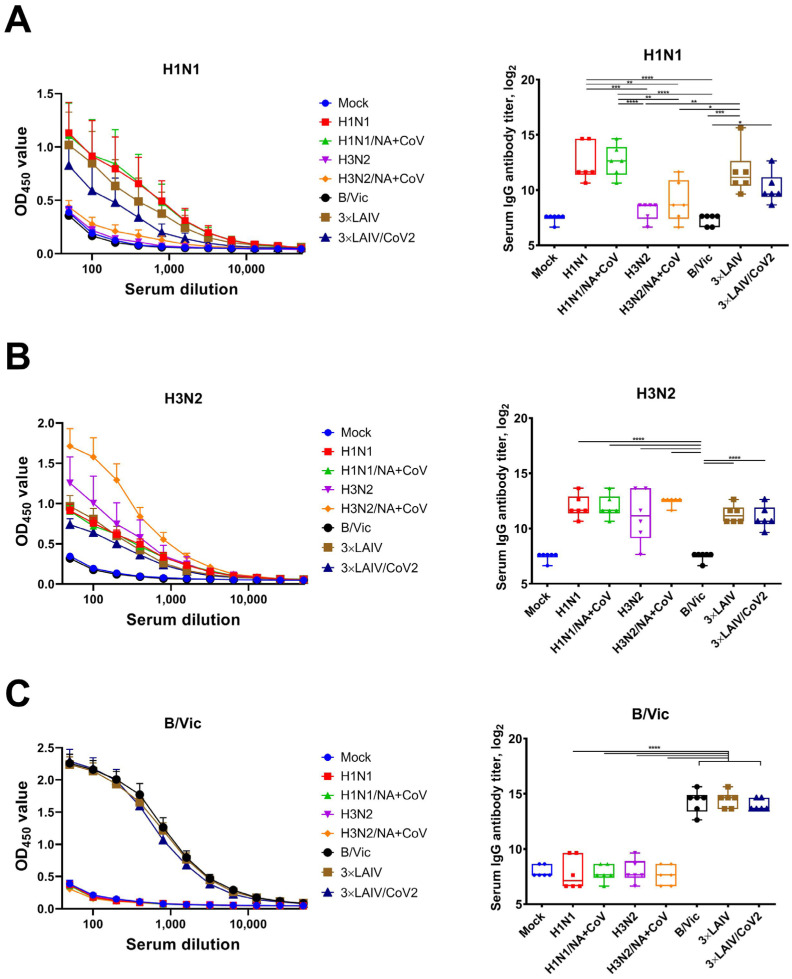
Serum IgG antibody responses to influenza viruses in immunized hamsters (ELISA with whole sucrose gradient-purified virus antigens). OD_450_ values (**left panel**) and endpoint serum IgG titers (**right panel**) in reaction with H1N1 antigens (**A**), H3N2 antigens (**B**) and B/Victoria antigens (**C**). Antibody levels were measured at day 42 of the study, after two doses of the vaccines. (****) *p* < 0.0001, (***) *p* < 0.001, (**) *p* < 0.01, (*) *p* < 0.05 (ANOVA with post hoc Tukey test).

Influenza-specific antibody immune responses were assessed at D42 of the study (3 weeks after the second dose). The studied vaccines stimulated IgG antibody response to the strain to which the animals were immunized (for monovalent controls) and to each component in trivalent formulations, both after the immunization with 3×LAIV/CoV2 and after the standard LAIV preparation (Figure 4). The immune response to the H1N1 component in both trivalent formulations was somewhat lower compared to the monovalent H1N1 vaccine (Figure 4A). Decreased immunogenicity of one of the components of multivalent formulations is usually due to strain characteristics and may vary from season to season depending on the recommended strain. Thus, decreased efficacy was described for the H1N1 component of the FluMist in 2013–2014 and 2015–2016 seasons [30,31,32,33], while in 2012–2013 poor immunogenicity was observed for the H3N2 component [34]. In our study, there were no differences in titers of antibodies to H3N2 and influenza B viruses after immunization with monovalent and trivalent vaccines (Figure 4B,C).

### 3.4. Protection Against Influenza Challenge

**Figure 5:**
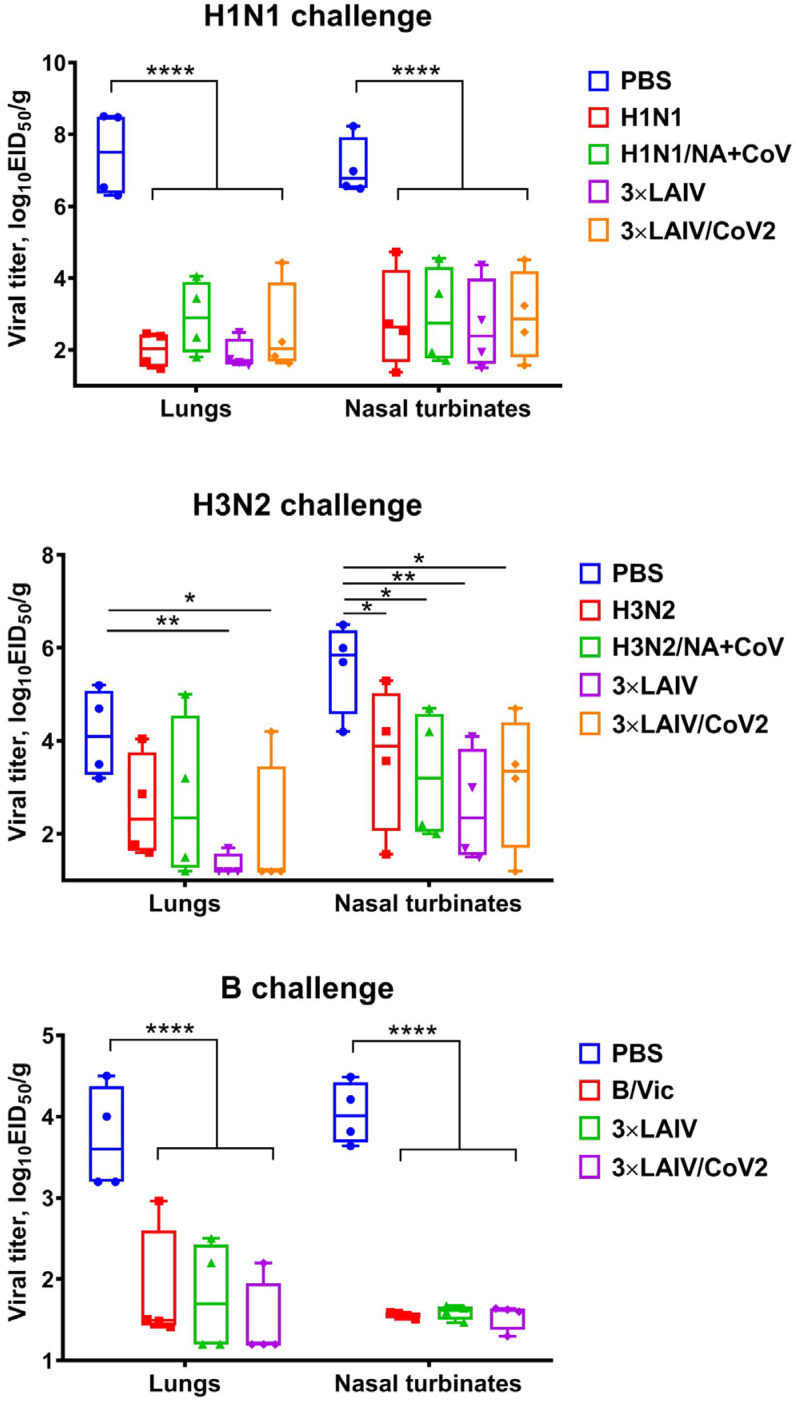
Protective efficacy of a modified trivalent LAIV and control monovalent and trivalent LAIVs against three seasonal influenza viruses in the Syrian hamster model. Titers of influenza A/H1N1 (**top panel**), A/H3N2 (**middle panel**) and type B viruses (**bottom panel**) in the lungs and nasal turbinates are shown. Animals in the placebo group received PBS. (****) *p* < 0.0001, (**) *p* < 0.01, (*) *p* < 0.05 (ANOVA with post hoc Tukey test).

On the third day after influenza challenge, lungs and nasal turbinates were harvested from immunized and control hamsters, where influenza virus titers were determined by limiting dilutions in eggs. As expected, homologous monovalent LAIVs significantly reduced virus replication both in the upper and the lower respiratory tract, with the exception of H3N2 challenge, where reduction in viral pulmonary titers compared to the mock group did not reach statistical significance, most likely due to the low number of animals in each group and the not intensive replication of this strain in the influenza-naïve hamsters (Figure 5). In the organs of animals immunized with the modified trivalent 3×LAIV/CoV2, which included the recombinant strains H1N1/NA+CoV2 and H3N2/NA+CoV2, as well as in the control group of trivalent 3×LAIV, significantly lower amounts of the infectious influenza viruses A/H1N1, A/H3N2 and B were detected than in the tissues of the animals that received the placebo preparation (Figure 5). These data confirm the ability of the trivalent modified vaccine to form protective immunity against the three seasonal influenza viruses.

Pathomorphological study revealed no significant changes in lung tissues of influenza-infected animals, even in the PBS group, which precluded the use of histologic data as a parameter for evaluating the protective effect of vaccines against influenza infection. Indeed, influenza infection in hamsters is mildly symptomatic, and the protective effect of vaccines is not usually assessed by histology [35,36].

### 3.5. Protection Against SARS-CoV-2 Infection

Although the inserted SARS-CoV-2 T-cell cassettes were designed to include HLA-restricted epitopes specific to humans, the cassette included prolonged regions of viral S and N proteins that could likely contain hamster-specific T-cell epitopes as well. Therefore, we checked whether our modified trivalent 3×LAIV/CoV2 could protect hamsters against a high dose of SARS-CoV-2 administered intranasally four weeks after the second vaccine dose.

**Figure 6:**
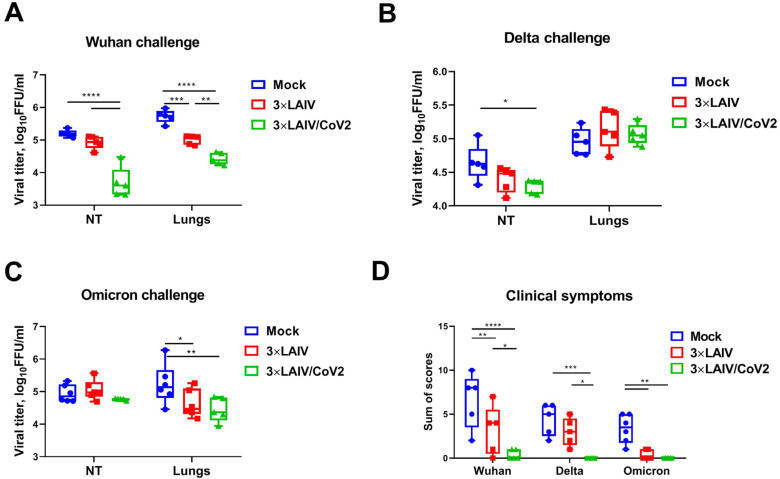
Protective efficacy of a modified trivalent LAIV and control monovalent and trivalent LAIVs against SARS-CoV-2 of three different antigenic lineages. Viral titers in nasal turbinates (NTs) and lung tissues on day 6 post-challenge are shown: (**A**) Wuhan strain; (**B**) Delta variant; (**C**) Omicron variant. (**D**) Sum of clinical symptom scores within 6 days of infection. (****) *p* < 0.0001, (***) *p* < 0.001, (**) *p* < 0.01, (*) *p* < 0.05 (ANOVA with post hoc Tukey test).

Lower amounts of infectious SARS-CoV-2 Wuhan virus were detected in the nasal turbinates of animals immunized with the modified 3×LAIV/CoV2 vaccine preparation than in the tissues of animals that received the classical 3×LAIV or placebo (Figure 6A). For the classical trivalent vaccine, there was also a decrease in Wuhan virus titers in the lungs, which may indicate a cross-reactivity of the induced immune response. For the Delta strain, a significant difference in viral titers was only observed in the NTs of 3×LAIV/CoV2-immunized hamsters compared to PBS (Figure 6B). Strikingly, despite a not very pronounced reduction in the Wuhan and Delta viral pulmonary replication in the vaccinated animals, the hamsters that received the modified trivalent vaccine showed a significantly less severe clinical picture of the disease after infection with SARS-CoV-2 than the control group of animals (Figure 6D). Of note, a decrease in the Omicron infectious titers in the lungs of animals immunized with both vaccine preparations was observed, also indicating the cross-protective potential of standard 3×LAIV (Figure 6C). These findings correlated with a similar decrease in clinical manifestation of disease after Omicron challenge (Figure 6D).

**Figure 7:**
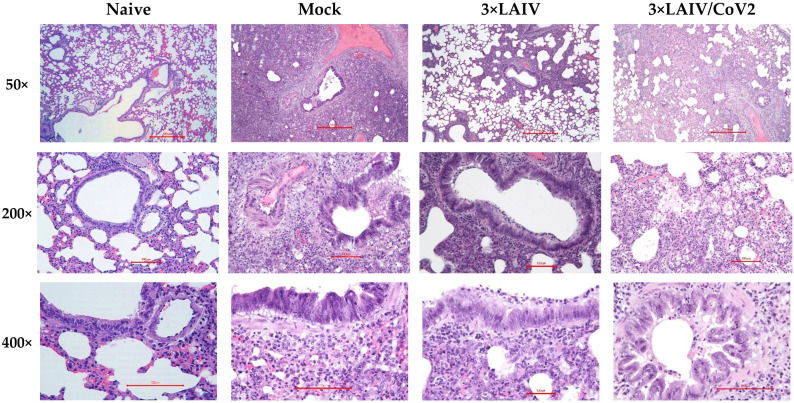
Histopathological assessment of protective effect of 3×LAIV/CoV2 against Wuhan challenge in a hamster model. Representative micrographs of hematoxylin–eosin-stained lung sections of animals on day 6 after challenge are shown using 50× (**upper panel**, scale bar: 500 µm), 200× (**middle panel**, scale bar: 100 µm) and 400× magnifications (**lower panel**, scale bar: 100 µm).

**Figure 8:**
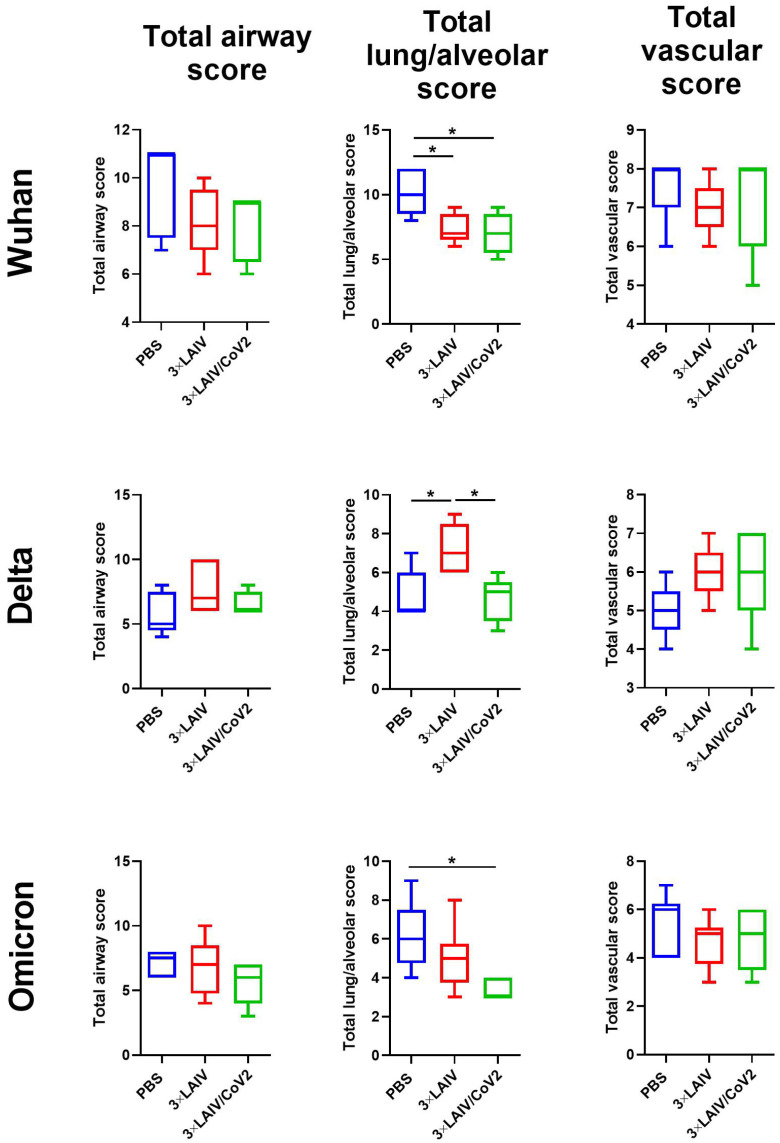
Semi-quantitative analyses of the airway, lung/alveolar and vascular damage to the lungs of immunized and control hamsters on day 6 after challenge with Wuhan (**upper panel**), Delta (**middle panel**) and Omicron (**lower panel**) SARS-CoV-2 variants. Data were analyzed by one-way ANOVA with Tukey’s post hoc multiple-analyses test. (*) *p* < 0.05.

Histological assessment of lung tissues collected from the SARS-CoV-2-challenged hamsters revealed that approximately 60% of the histological slides in the mock group infected with the Wuhan variant were characterized by more than 50% damage to lung and vascular tissues. Furthermore, this group had most severe alveolar inflammation with loss of normal septal histoarchitectonics (Figure 7). In addition, there was a more frequent desquamation of the necrotic bronchiolar epithelium into the bronchi lumen compared with the other control groups. In the 3×LAIV-immunized group, lung pathology was less pronounced compared to the control animals. In particular, alveoli were affected in a smaller percentage and at a lower severity. Bronchioles as well as vessels were affected mainly in the apical segments of the lungs. A further significant reduction in total lung/alveolar score values was achieved in the 3×LAIV/CoV2 group (Figure 8), in which there was almost a complete absence of type II pneumocyte hyperplasia compared to the control group. Although the lung histoarchitectonics was not comparable to that of the intact hamsters’ lungs, this group was the least affected by Wuhan challenge.

In the Delta and Omicron infection control groups, the histopathological pictures were similar: moderate (25–50%) lung and vascular damage was observed segmentally, mainly in the apical lung segments (Figures S1 and S2). Semi-quantitative scoring of lung pathology revealed significant differences in all criteria between the Wuhan control and the other two control groups (Figure 8). There were milder bronchiolitis and a smaller percentage (10–25% of sectioned airways) of bronchiolar epithelial hyperplasia compared to the Wuhan control group. Lower pathology scores for the Delta variant did not allow us to identify any significant differences in score values between the mock and the 3×LAIV/CoV2 groups. However, in Omicron challenge, a quantitative analysis of lung pathology revealed a significant reduction in total lung/alveolar score values in the 3×LAIV/CoV2 group relative to the control (Figure 8). The alveoli architectonics was comparable to that of naïve hamsters, as the spread of inflammatory infiltration was minimal and did not affect interalveolar septa.

### 3.6. Cell-Mediated Immune Responses to Influenza and SARS-CoV-2 Antigens

**Figure 9:**
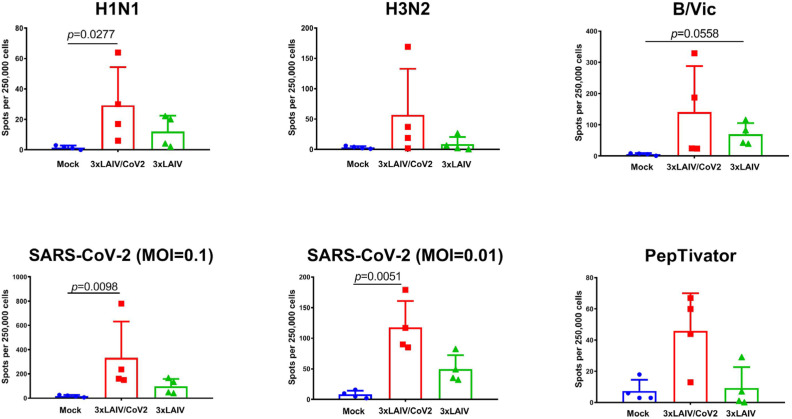
Levels of IFNγ-secreting cells in splenocytes of immunized Syrian hamsters on day 6 after infection with hCoV-19/Russia/StPetersburg-3524/2020 (line B.1, Wuhan). Isolated splenocytes were stimulated in vitro with influenza viruses (**upper panel**) and live SARS-CoV-2 or PepTivator (**lower panel**), followed by quantification of IFNγ-secreting cells using the Hamster IFN-γ ELISpot Plus kit.

Cell-mediated immunity was assessed in splenocytes of hamsters euthanized on day 6 after challenge with the Wuhan and Delta SARS-CoV-2 variants. Stimulation of immune cells in vitro with whole influenza viruses revealed higher levels of IFNγ-secreting cells in 3×LAIV/CoV2-immunized animals than in hamsters immunized with classical seasonal 3×LAIV (Figure 9 and Figure S3), which is in concordance with our previous findings on the augmented influenza-specific T-cell responses to recombinant LAIVs expressing foreign immunodominant T-cell epitopes [37]. Additionally, it was shown that during infection with SARS-CoV-2 of two genetic lineages, a more pronounced stimulation of SARS-CoV-2-specific splenocytes was detected in the group of Syrian hamsters immunized with the modified trivalent vaccine preparation compared to the classical trivalent LAIV (Figure 9 and Figure S3).

In summary, the experiments in Syrian hamsters showed that the modified trivalent vaccine preparation, 3×LAIV/CoV2, containing two recombinant vaccine strains, H1N1/NA+CoV2 and H3N2/NA+CoV2, encoding additional T-cell epitopes of SARS-CoV-2, and the classical type B LAIV strain, stimulates humoral and cellular immunity to the three seasonal circulating influenza viruses, as well as T-cell immunity to SARS-CoV-2. Immunity formed in response to immunization is able to protect animals from infection caused by three influenza virus variants as well as several genetically diverse variants of SARS-CoV-2.

## 4. Discussion

The idea of developing a bivalent vaccine to prevent both influenza and SARS-CoV-2 infections through annual influenza immunization campaigns is a promising way to increase immunization coverage. A list of such experimental vaccine candidates has been recently developed, including VSV-based [38,39], adenovirus-vectored [40,41], influenza VLP-based [42], lentivirus VLP-based [43] and mRNA-based [44] vaccines, as well as a variety of preparations using the influenza virus as a vector [45,46,47,48,49,50,51,52,53,54]. In some formulations, broad anti-influenza protection is expected due to the use of conserved influenza fragments. Thus, the hemagglutinin stalk-based strategy was used in adenovirus-vectored vaccine [40], chimpanzee AdV [41] and chimeric recombinant protein preparations [55]. Similarly, the M2e-targeted strategy was used for a VSV-based candidate [38,39]. Despite the promising idea of the development of a universal influenza vaccine, at present, annual influenza vaccinations are still carried out using trivalent or quadrivalent preparations, which are updated twice a year by the World Health Organization. Unlike the vaccines described above, our candidate vaccine is a trivalent live attenuated influenza vaccine with the possibility of regular updating of composition that will provide protection against all seasonal influenza viruses according to the standard protocol. At the same time, the vaccine does not contain structural SARS-CoV-2 proteins, such as RBD, and does not induce S-specific antibodies. Rare adverse events such as vascular and coagulation dysfunction, which have been observed after mRNA vaccine administration, have been associated with the SARS-CoV-2 Spike protein (due to the presence of molecular mimicry of human protein phenomena) [56]. It should be noted that LAIV strains based on MDVs, A/Leningrad/17 and B/USSR/60/69 have been used in clinical practice for about 40 years, with no detectable reversion to the pathogenic form, which is attributed to the control of the attenuated phenotype by a number of specific mutations in various genes [57,58]. Virus shedding from the vaccine studied in non-human primates was extremely low even after administration of the first dose [7], confirming that the modification did not dramatically increase the transmissibility of LAIV strains, but further studies are needed to establish the reproducibility of this effect in different animal models. Cold-adapted LAIV strains have previously been shown to have reduced transmissibility (reviewed in [59]), which also indicates vaccine safety.

Since all influenza/COVID-19 bivalent vaccines are in the early stages of development and have limited protective potential against influenza, the idea of coadministration of licensed vaccines against influenza and SARS-CoV-2 is being widely explored. In October 2021, WHO published official guidance regarding coadministration of several COVID-19 vaccines with influenza vaccines [60]. In the season following the registration of the first SARS-CoV-2 vaccines for human use, a significant proportion of people were afraid of administering two vaccines at the same time, and this was banned in some countries, but studies have shown that people who were negative about coadministration of the vaccines responded favorably to the idea of developing a combination product specifically designed and studied as a bivalent vaccine [61,62]. A number of clinical trials investigating the coadministration of COVID-19 vaccines with seasonal influenza vaccines [63,64,65,66,67,68] have reduced the refusal rate for coadministration of vaccines against these two pathogens; however, almost every fifth person is not ready to receive two vaccines at the same time, not for objective reasons (allergy to one of the drugs or unavailability of one of the drugs), but because of concerns about increased side effects and additional pain; these patients prefer separate administration of vaccines, spaced out over time [64], which leads to lower vaccination coverage against these infections. In this context, our candidate vaccine is particularly promising because it is a complete trivalent influenza vaccine with a modified genome, i.e., it is a specially designed formulation for use as a single combination vaccine. In addition, the non-invasive route of administration also increases the attractiveness of this vaccine in a number of cases.

To address this problem, mRNA vaccine manufacturers are combining a mixture of different vaccines in a single shot and are studying these formulations as a new commercial product. Moderna’s candidate mRNA-1083 is a combination of an influenza quadrivalent vaccine (encoding HAs of the H1N1, H3N2, B/Vic and B/Yam strains) and mRNA-1283 (SARS-CoV-2 NTD + RBD), and Phase 2 results were published in 2022 [69]. According to a press release, the vaccine was immunogenic and effective in a Phase 3 clinical trial (NCT06097273) [70]. Recently, the results of a study of a similar formulation developed by Pfizer+BioNTech (NCT06178991) were announced [71]; earlier, the results of a study of coadministration of BNT162b2 with an influenza vaccine (NCT05310084) were published [65]. Of note, emerging data suggest that repeated administration of mRNA COVID-19 vaccines resulted in antibody class switch to IgG4 with increased tolerance to vaccine antigens [72,73], which may significantly affect vaccine efficacy because antibodies of this class have very weak capability to activate antibody-dependent immune effector responses [74]. These data suggest that the use of combined mRNA vaccines in annual vaccination campaigns may have unexpected consequences for the quality of induced antibodies against both influenza viruses and SARS-CoV-2. The vaccine we developed is fundamentally different in its mechanism of action from mRNA vaccines; in addition, the SARS-CoV-2-specific component included in the vaccine is not aimed at inducing antibodies and undergoes intracellular processing for targeted stimulation of the T-cell immune response. The cassette contains fragments of the N protein and a conserved region of the Spike protein (not RBD), and we tested the candidate vaccine for the absence of induction of an antibody response to SARS-CoV-2 in our previous experiments [6]. For the influenza component, subtyping of antibody classes was performed in our studies of different LAIV-based vaccine candidates in mice. Thus, the balanced IgG1/IgG2a class antibodies were elicited by intranasal immunization, and the leading role of T-cell immunity in protection, especially tissue-resident memory T-cells, was demonstrated in this animal model [37,75]. It is worth noting that the early studies of human serum IgG responses to LAIV demonstrated a low abundance of IgG4 subclass antibodies in response to LAIV administration in different age groups [76,77].

The use of live influenza vaccines as carriers for intranasal administration of combination vaccines has several advantages over inactivated preparations administered intramuscularly. Despite lower serum antibody responses [78], LAIV has comparable efficacy due to stimulation of mucosal immunity [79,80,81], formation of long-lived tissue memory T-cells [82] and the fact that serum antibodies formed in response to LAIV are more functional than those formed after inactivated vaccine administration [80]. Because of the immunity formation patterns, LAIV is recommended as a product of choice during the onset of an influenza pandemic [59,83]. Thus, LAIV stimulates innate immunity, one of the factors of non-specific defense against influenza [84], and also forms a mucosal barrier with cross-reactive specific immune factors, which prevents the spread of infection [79,82,85,86,87]. Studies have demonstrated cross-reactivity of tissue-resident memory T-cells against different influenza strains [86]. Finally, long-term use of seasonal A/Leningrad-based LAIV has demonstrated the safety and effectiveness of this preparation in various age groups [59].

One of the limitations of this study is that we did not evaluate the longevity of immune responses induced by influenza and SARS-CoV-2 antigens, which is planned for our future experiments. Importantly, our vaccine is biologically identical to classical LAIV viruses and has exactly the same pathways of antigen delivery and presentation. Therefore, it is reasonable to suggest that the data accumulated for LAIVs in multiple clinical studies can be translated to this modified 3×LAIV/CoV2 vaccine prototype. Specifically, studies in children found that LAIVs could elicit durable humoral and cell-medicated immune responses which could be directly measured six to twelve months after immunization [16]. In addition, studies of H5N1/H5N2 pandemic LAIVs in healthy adults have shown that despite very low antibody titers after immunization, the long-lasting immunity could be unmasked by administration of an inactivated H5N1 influenza vaccine several years later, which promoted the rapid formation of high-titer, broadly reactive antibodies [88,89]. Regarding the durability of SARS-CoV-2-induced T-cell responses, virus-specific T-cells, in contrast to Spike-specific antibodies, persisted for a long period after recovery from COVID-19 [90,91]. Thus, we can hypothesize that our vaccine viruses, which deliver immunogenic T-cell epitopes directly to target cells in the respiratory tract, may also induce durable T-cell immunity to SARS-CoV-2.

Another limitation of this study is the lack of immunophenotyping of the hamsters’ cells in assessing T-cell responses. Detailed studies of the immune response are usually performed in a mouse model because of the availability of specific reagents [40,92]. However, mice are not a suitable model for SARS-CoV-2 infection or H3N2 influenza infection. In our study, we focused on vaccine safety and protective potential against both infections; therefore, hamsters were chosen as model animals. For the H3N2 vaccine component studied here, the balance of T-cells in peripheral blood has been previously assessed in a rhesus macaque model [7]. At the next stage, we plan to conduct more detailed studies of the immune response to the vaccine in small animals to identify specific correlates of protection.

We previously developed combined vaccine candidates, where SARS-CoV-2 T-cell cassettes were inserted into the genomes of H7N9 [6] or H3N2 [7] LAIV strains. Both modified vaccine candidates demonstrated enhanced immunogenicity to influenza virus antigens compared to control non-modified LAIV strains in animal models; and for the H7N9-based strain, we studied protective efficacy against two infections in hamsters [6,7]. In these proof-of-concept studies, the SARS-CoV-2 cassette was incorporated into NA open reading frames of different influenza strains. Since the cassette could be inserted into different influenza subtypes with reproducible in vitro characteristics and the modified strains have demonstrated safety and efficacy in various experiments, we decided to test this strategy in an experiment with a standard trivalent influenza LAIV formulation with SARS-CoV-2 inserts. This design allows the vaccine to be updated each season, maintaining maximum protective effectiveness against influenza while generating baseline immunity to conserved parts of the coronavirus. The current study confirmed that immunization of Syrian hamsters with a mixture of three seasonal LAIV strains, two of which encoded the SARS-CoV-2 cassette, induced high levels of influenza-specific antibody responses and efficiently protected animals against replication of homologous wild-type influenza viruses in the respiratory tract, and no negative effects of the H1N1 or H3N2 LAIV strain modifications were noted when this modified vaccine was compared to the classical trivalent LAIV formulation.

It is important to note that administration of relatively high doses of modified and standard vaccine strains, both alone and in a mixture of three components, had no effect on the health status or behavior of the animals, while various laboratory blood parameters also did not change during immunization, indicating a high degree of vaccine safety when administered intranasally. In addition, there was no ADE effect when immunized animals were infected with live SARS-CoV-2 at a sufficiently high dose of 5 lgTCID_50_. Although we observed some protective effect of our modified trivalent 3×LAIV/CoV2 vaccine against SARS-CoV-2 of various lineages, these data are not directly transferable to humans, reflecting a common problem in evaluating human T-cell vaccines in animal model experiments. There are no established correlates of protection for such vaccines and no protocols for effective in vitro evaluation. The vaccine developed should be evaluated in clinical trials, as was done for the dNS1-RBD influenza vector vaccine [46,93].

Nevertheless, the cross-reactive potential of the vaccine against different SARS-CoV-2 strains is expected due to the presence of prolonged SARS-CoV-2 N fragments in the cassette: this protein is conserved among SARS-CoV-2 strains and is one of the main targets of the T-cell response, and the fragments to be incorporated were selected based on experimentally established epitope contents [94,95,96,97,98]. In future studies, we plan to develop the principles of cassette updating, whereas in the present work, we studied the interference of LAIV strains encoding SARS-CoV-2 cassettes within a trivalent LAIV formulation. Furthermore, our future studies will aim to evaluate the effects of age and sex, as well as pre-immune status, on the elicited immune responses and the vaccine-induced protection against influenza and SARS-CoV-2 strains and on the protective potential of a single-dose immunization regimen. For example, 66-week-old Syrian hamsters were successfully immunized with a modified Vaccinia virus Ankara (MVA)-vectored COVID-19 vaccine and demonstrated robust antibody and T-cell immune responses to the inserted SARS-CoV-2-stabilized Spike protein [99].

Finally, it should be noted that in this work we studied vaccine candidates grown in eggs. At the same time, we showed that the modified viruses can also replicate actively in MDCK cells, which determines the possibility of their industrial production in these cells using bioreactors. Strains produced in MDCK cells are known to be less susceptible to mutagenesis of surface antigens, resulting in the induction of antibodies that recognize circulating viruses better than egg-grown vaccines, and, as a result, cell-based vaccines are more effective in protecting people against seasonal influenza than egg-based vaccines [100,101,102]. In addition, the ability to produce vaccine viruses in cells instead of chicken embryos will allow production levels to remain high even in the event of an avian influenza pandemic when the availability of eggs for production is disrupted. And our future work will focus on the development of modified trivalent LAIVs grown on MDCK cells, specifically studying their stability after multiple passages in this substrate, as well as a detailed evaluation of immunogenicity and protective activity against all three strains of seasonal influenza and against different coronavirus variants.

## 5. Conclusions

We used a licensed seasonal trivalent live attenuated influenza vaccine (3×LAIV) as a basis for the development of a modified 3×LAIV/CoV2 vaccine, where H1N1 and H3N2 LAIV strains encoded an immunogenic cassette enriched with conserved T-cell epitopes of SARS-CoV-2, whereas the B/Victoria lineage LAIV strain was unmodified. Experiments in a golden Syrian hamster model revealed that both the classical 3×LAIV and 3×LAIV/CoV2 vaccine compositions induced similar levels of serum antibodies specific to all three influenza strains, which resulted in comparable levels of protection against challenge with either influenza strain. Protection against SARS-CoV-2 challenge was more pronounced in the 3×LAIV/CoV2-immunized hamsters compared to the classical LAIV group. These data were accompanied by the higher magnitude of virus-specific cellular responses detected by ELISPOT in the modified trivalent LAIV group. Therefore, the modified trivalent live attenuated influenza vaccine encoding the T-cell epitopes of SARS-CoV-2 can be considered a promising tool for combined protection against seasonal influenza and COVID-19.

## Figures and Tables

**Table 1:** Growth characteristics of classical and chimeric LAIV viruses in vitro and in vivo.

LAIV Virus	Viral Titers in Eggs, lgEID_50_/mL			Viral Titers in MDCK Cells, lgTCID_50_/mL	Viral Titers in Hamster Tissues, lgEID_50_/g		
	26 °C	33 °C	40 °C		NT ^1^	Lung	Trachea
H1N1	7.5	9.6	<1.2	7.0	4.9 ± 0.5	3.4 ± 0.5	2.0 ± 0.3
H1N1/NA+CoV	6.8	8.5	<1.2	7.0	4.1 ± 0.6	1.8 ± 0.2	2.1 ± 0.1
H3N2	6.5	9.0	<1.2	8.4	3.2 ± 1.9	1.8 ± 0.1	2.1 ± 0.2
H3N2/NA+CoV	6.7	8.5	<1.2	8.6	3.1 ± 0.9	2.0 ± 0.4	2.2 ± 0.1
B/Victoria	7.8	9.9	<1.2	9.5	4.3 ± 1.0	2.2 ± 0.4	3.6 ± 0.6

^1^ NT: nasal turbinates.

## Data Availability

The data presented in this study are available on request from the corresponding author.

## Supplementary Materials

The following supporting information can be downloaded at: https://www.mdpi.com/article/10.3390/vaccines12121300/s1, Figure S1. Histopathological assessment of protective effect of 3×LAIV/CoV2 against Delta challenge in a hamster model; Figure S2. Histopathological assessment of protective effect of 3×LAIV/CoV2 against Omicron challenge in a hamster model; Figure S3. Levels of IFNγ-secreting cells in splenocytes of immunized Syrian hamsters on day 6 after infection with hCoV-19/Russia/SPE-RII-32759S/2021 (line B.1.617.2, Delta); Table S1. Scoring for histopathological changes in lung tissues due to SARS-CoV-2 infection; Table S2. Clinical blood test results of hamsters, days 41–42 of the experiment; Table S3. Blood biochemical parameters of hamsters, days 41–42 of the experiment; Table S4. The results of estimation of mass factors of internal organs of hamsters, day 42 of the experiment; Table S5. Summary of pathological changes in hamsters’ organs and tissues found during necropsy on day 42 of the study.
