# Supplementary material for: Safety, Immunogenicity and Protective Activity of a Modified Trivalent Live Attenuated Influenza Vaccine for Combined Protection Against Seasonal Influenza and COVID-19 in Golden Syrian Hamsters
Source: Vaccines (Basel). 2024 Nov 21;12(12):1300. doi: 10.3390/vaccines12121300 (PMC11679497; doi:10.3390/vaccines12121300)
Supplement: Supplementary file 1 [file vaccines-12-01300-s001.zip › vaccines-3318321-supplementary.pdf]

Supplementary materials

Safety, immunogenicity and protective activity of a modified trivalent live attenuated influenza vaccine for combined protection against seasonal influenza and COVID-19 in golden Syrian hamsters

Ekaterina Stepanova [1], Victoria Matyushenko [1], Daria Mezhenskaya [1], Ekaterina Bazhenova [1], Tatiana Kotomina [1], Alexandra Rak [1], Svetlana Donina [1], Anna Chistyakova [1], Arina Kostromitina [1], Vlada Novitskaya [1], Polina Prokopenko [1], Kristina Rodionova [1], Konstantin Sivak [2], Kirill Kryshen [3], Valery Makarov [3], Larisa Rudenko [1], and Irina Isakova-Sivak [1]*

Table S1. Scoring for histopathological changes in lung tissues due to SARS-CoV-2 infection

Parameter assessed	Score	Criteria for scoring
**Damage to the airways (total airway score)**		
% airway affected	0	none
	1	<10%,
	2	10–25%
	3	25–50%
	4	50+%
airway severity	0	minimal peribronchial/peribronchiolar mononuclear infiltrates
	1	mild peribronchitis/bronchiolitis
	2	mild to moderate mononuclear to mixed peribronchiolitis/lumens contain low numbers of inflammatory cells/multifocal single cell necrosis of airway epithelium
	3	moderate to marked mixed peribronchiolitis/large foci of bronchiolar epithelial necrosis/occasional atypical or multinucleated cells
	4	marked bronchiolitis and widespread epithelial necrosis +/− rupture of bronchiolar epithelium, and/or frequent atypical/syncytial cells
bronchiolar epithelial hyperplasia	0	none
	1	sporadic bronchiolar epithelial hyperplasia < 10% section's airways
	2	mild to moderate bronchiolar epithelial hyperplasia 10–25% section's airways
	3	widespread bronchiolar epithelial hyperplasia and/or multinucleated syncytial cells taking up 25+% section's airways
**Damage to the lung parenchyma (total lung/alveolar score)**		
% alveoli affected	0	none
	1	<10%,
	2	10–25%
	3	25–50%
	4	50+%
alveolar severity	0	within normal margins (rare/minimal peribronchial/peribronchiolar mononuclear infiltrates)
	1	mild peribronchiolar primary mononuclear inflammatory infiltrates, extending into adjacent alveolar septa/spaces
	2	mild to moderate, mononuclear to mixed inflammation (>3 cell layer), expands alveolar septa or spaces/obscures normal septal architecture
	3	moderate mixed interstitial inflammation, and/or alveolar damage characterized by type I pneumocyte necrosis/loss with replacement by hemorrhage, fibrin, edema, necrotic debris (reminiscent of hyaline membranes) and/or scattered atypical/syncytial cells
	4	marked alveolar inflammation (mixed), alveolar septal damage (all above) + loss of normal septal architecture with frequent syncytial cells)
type II pneumocyte hyperplasia	0	none
	1	scattered type II pneumocyte hyperplasia taking up <10% of the section
	2	mild to moderate type II pneumocyte hyperplasia taking up 10–15% + atypical multinucleated cells
	3	widespread type II pneumocyte hyperplasia taking up 25+% of the section
alveolar edema/protein/hemorrhage/fibrin	0	none
	1	present
**Damage to the vascular bed (total vascular score)**		
% vessels affected	0	none
	1	<10%,

	2	10–25%
	3	25–50%
	4	50+%
vascular/perivascular lesions	0	none
	1	multifocal perivascular edema/mild mononuclear perivascular inflammation
	2	moderate mononuclear to mixed perivascular inflammation, edema or fibrin with leukocytes occasionally transmigrating the vessel wall/multifocal endotheliitis
	3	severe mixed perivascular infiltration, expanding/replacing vessel wall and/or marked frequent endotheliitis
necrotizing vasculitis/thrombi	0	none
	1	present

Table S2. Clinical blood test results of hamsters, day 41-42 of the experiment [A], M±SEM, n=6

Parameter	Group							
	Mock	H1N1-NA/CoV	H1N1	H3N2-NA/CoV	H3N2	B/Vic	3×LAIV/CoV2	3×LAIV
RBC, 10^{12}/L	10,1±0,17	9,9±0,23	9,3±0,23	10,1±0,25	9,3±0,18	9,3±0,11	10,0±0,27	9,2±0,16
HGB, g/L	163,3±1,74	164,7±3,11	152,2±3,37	162,8±4,09	156,0±2,73	155,0±2,39	162,3±4,95	153,0±2,70
MCH, pg	16,3±0,12	16,7±0,13	16,4±0,14	16,2±0,11	16,7±0,14	16,6±0,12	16,3±0,11	16,6±0,07
MCHC, g/L	357,2±2,57	357,5±2,20	356,5±1,34	355,7±2,42	366,0±1,51 [B]	363,7±1,41	358,3±0,42	362,0±1,41
HCT, %	0,46±0,008	0,46±0,010	0,43±0,008	0,46±0,010	0,43±0,008	0,43±0,006	0,45±0,014	0,42±0,007
PLT, 10^9/L	817,7±34,06	691,2±43,76	639,2±26,18 [B]	610,5±31,96 [B]	689,0±26,19	600,0±48,81 [B]	608,3±55,02 [B]	692,5±13,61
MPV, fL	7,4±0,18	7,5±0,09	7,2±0,19	8,1±0,34	7,3±0,23	7,8±0,21	7,5±0,24	7,7±0,24
WBC, 10^9/L	6,9±0,55	7,9±0,49	7,1±0,59	7,9±0,95	5,8±0,22	4,9±0,54	5,2±0,39	3,8±0,32 [B]
PCT, %	0,61±0,038	0,52±0,027	0,46±0,016 [B]	0,49±0,023	0,50±0,016	0,47±0,042 [B]	0,45±0,031 [B]	0,54±0,025
MCV, fL	45,5±0,28	46,8±0,27	46,1±0,43	45,5±0,23	45,8±0,27	45,7±0,20	45,4±0,30	45,8±0,31
LYM, %	68,1±0,67	63,9±1,76	66,4±1,44	67,7±2,42	61,2±3,35	65,6±1,33	64,5±3,81	66,2±2,19
MON, %	5,8±0,62	4,9±0,48	4,6±0,24	4,1±0,53	5,0±0,23	5,3±0,43	4,8±0,50	4,9±0,58
GRA, %	26,2±0,80	31,2±1,93	29,0±1,44	28,2±2,33	33,9±3,28	29,1±1,20	30,7±3,62	28,9±1,64

1 – [A] – the data were normally distributed (p>0,05, Shapiro-Wilk test) and the assumption of homogeneity of variances was satisfied (p>0,05, Brown-Forsite test) One-way ANOVA revealed the effect of "Group" factor to WBC, HGB, RBC, HCT, PLT, MCH, MCHC, PCT (p<0,05);

2 – [B] – the statistically significant differences compared with control (p<0,05, Tukey test).

Table S3. Blood biochemical parameters of hamsters, day 41-42 of the experiment [A], M±SEM, n=6

Parameter	Group							
	Mock	H1N1-NA/CoV	H1N1	H3N2-NA/CoV	H3N2	B/Vic	3× LAIV/CoV2	3×LAIV
ALB , g/L	19,1±0,47	20,4±0,37	21,2±0,61	21,4±0,40	21,5±0,55	23,1±1,04 [B]	21,6±0,65	21,3±0,72
Total protein (TP), g/L	50,5±1,89	54,6±0,82	53,1±2,29	56,2±1,37	56,1±1,01	59,6±2,80	55,8±1,84	55,5±1,80
GLOB , g/L	31,5±1,59	34,2±0,49	32,0±1,70	34,8±0,98	34,7±0,48	36,6±1,79	34,3±1,39	34,2±1,14
ALB/GLOB	0,61±0,029	0,6±0,006	0,67±0,017	0,62±0,007	0,62±0,009	0,63±0,007	0,63±0,021	0,62±0,010
ALT, U/L	91,4±12,98	88,9±8,72	81,9±3,26	75,3±4,09	70,3±2,13	86,3±6,16	73,2±5,22	84,2±3,04
AST, U/L	89,2±14,08	86,4±6,91	70,3±5,41	84,3±6,90	68,8±9,04	87,4±9,98	72,0±7,97	74,1±8,70
CHOL, mmol/L	1,88±0,179	1,67±0,164	1,82±0,155	2,17±0,149	2,3±0,127	2,28±0,416	2,68±0,138	2,33±0,079
CREA, µmol/L	41,5±1,4	49,6±4,95	53,0±3,76	61,3±3,85	47,3±4,50	72,3±15,32	56,1±9,45	51,9±7,63
TG, mmol/L	1,08±0,100	1,14±0,111	1,09±0,106	1,44±0,14	1,99±0,232	1,80±0,145 [B]	2,03±0,193	2,29±0,148
BUN, mmol/L	7,2±0,37	8,3±0,45	7,9±0,35	8,1±0,26	6,3±0,24 [B]	9,8±0,77 [B]	8,2±0,43 [B]	6,4±0,28 [B]
BIL-T, µmol/L	1,7±0,36	1,6±0,25	1,6±0,31	2,4±0,25	2,6±0,45	1,6±0,25	2,6±0,25	1,7±0,21
Glucose, mmol/L	5,9±0,26	5,5±0,23	6,8±0,30	6,0±0,16	5,9±0,19	6,7±0,45	6,1±0,30	6,9±0,30

1 – [A] – the data were normally distributed (p>0,05, Shapiro-Wilk test) and the assumption of homogeneity of variances was satisfied (p>0,05, Brown-Forsite test) One-way ANOVA revealed the effect of “Group” factor to ALB, CHOL, TG, BUN, BIL-T (p<0,05);

2 – [B] – the statistically significant differences compared with control (p<0,05, Tukey test).

Table S4. The results of estimation of mass factors of internal organs of hamsters, day 42 of the experiment, % to body weight, M±SEM, n=6

Parameter	Group							
	Mock	H1N1-NA/CoV	H1N1	H3N2-NA/CoV	H3N2	B/Vic	3×LAIV/CoV2	3×LAIV
Heart [A]	0,50±0,029	0,47±0,014	0,49±0,015	0,54±0,043	0,46±0,016	0,52±0,013	0,65±0,123	0,49±0,027
Lungs with trachea [B]	0,73±0,079	0,64±0,018	0,67±0,027	0,60±0,014	0,65±0,026	0,72±0,045	0,83±0,126	0,59±0,027
Thymus [A]	0,085±0,0143	0,077±0,007	0,086±0,0053	0,073±0,0126	0,072±0,0146	0,072±0,0162	0,091±0,0098	0,049±0,0028
Liver [A]	4,37±0,183	3,66±0,055	3,56±0,090 [C]	3,47±0,120 [C]	3,83±0,070	4,27±0,162	4,08±0,279	4,14±0,255
Spleen [A]	0,10±0,014	0,14±0,011	0,13±0,007	0,11±0,007	0,14±0,004	0,14±0,020	0,13±0,015	0,10±0,005
Kidney [B]	1,00±0,054	0,98±0,023	0,94±0,013	0,94±0,033	1,01±0,019	1,07±0,048	1,01±0,052	0,95±0,040
Adrenal glands [A]	0,028±0,0028	0,031±0,0016	0,037±0,0030	0,046±0,0061 [C]	0,034±0,0011	0,042±0,0028	0,035±0,0027	0,039±0,0044
Brain [A]	1,10±0,068	0,88±0,045	0,96±0,040	1,00±0,102	0,85±0,034	1,02±0,051	1,01±0,038	0,92±0,034
Ovaria [A]	0,07±0,007	0,06±0,007	0,07±0,007	0,07±0,004	0,06±0,003	0,07±0,003	0,06±0,005	0,07±0,006

1 – [A] – the data were normally distributed (p>0,05, Shapiro-Wilk test) and the assumption of homogeneity of variances was satisfied (p>0,05, Brown-Forsite test) One-way ANOVA revealed the effect of "Group" factor to Liver and Adrenal glands mass factors;

2 – [B] – the data were normally distributed (p>0,05, Shapiro-Wilk test) and the assumption of homogeneity of variances was not satisfied (p<0,05, Brown-Forsite test) One-way ANOVA with Welch's correction have not revealed the effect of "Group" factor (p>0,05);

3 – [C] – the statistically significant differences compared with control (p<0,05, Tukey test).

Table S5. Summary of pathological changes in hamsters' organs and tissues found during necropsy on day 42 of the study

Study group	Description of changes	Number of animals affected
PBS	Hemorrhage in the lungs	1/6
	Pulmonary hemorrhage, distelectasis	3/6
H1N1 LAIV	Pulmonary hemorrhage, distelectasis	1/6
H1N1/NA+CoV2 LAIV	Dilation of the renal tubules	1/6
	Pulmonary hemorrhage, distelectasis	2/6
H3N2 LAIV	Hemorrhage in the lungs	1/6
	Dilation of the renal tubules	1/6
	Pulmonary hemorrhage, distelectasis	1/6
H3N2/NA+CoV2 LAIV	Hemorrhage in the lungs	1/6
	Pulmonary hemorrhage, distelectasis	2/6
B LAIV	Ovarian cyst	1/6
	Dilation of the renal tubules	2/6
	Pulmonary hemorrhage, distelectasis	3/6
3×LAIV	Pathological formation in liver	1/6
	Pathological formation in lungs	1/6
	Liver polycystic disease	1/6
	Ovarian cyst	1/6
	Pulmonary hemorrhage, distelectasis	2/6
	Abscessing pneumonia	1/6
3×LAIV/CoV2	Hemorrhage in the lungs	1/6
	Lung enlargement, foamy fluid, hyperemia	1/6
	Pulmonary hemorrhage, distelectasis	2/6

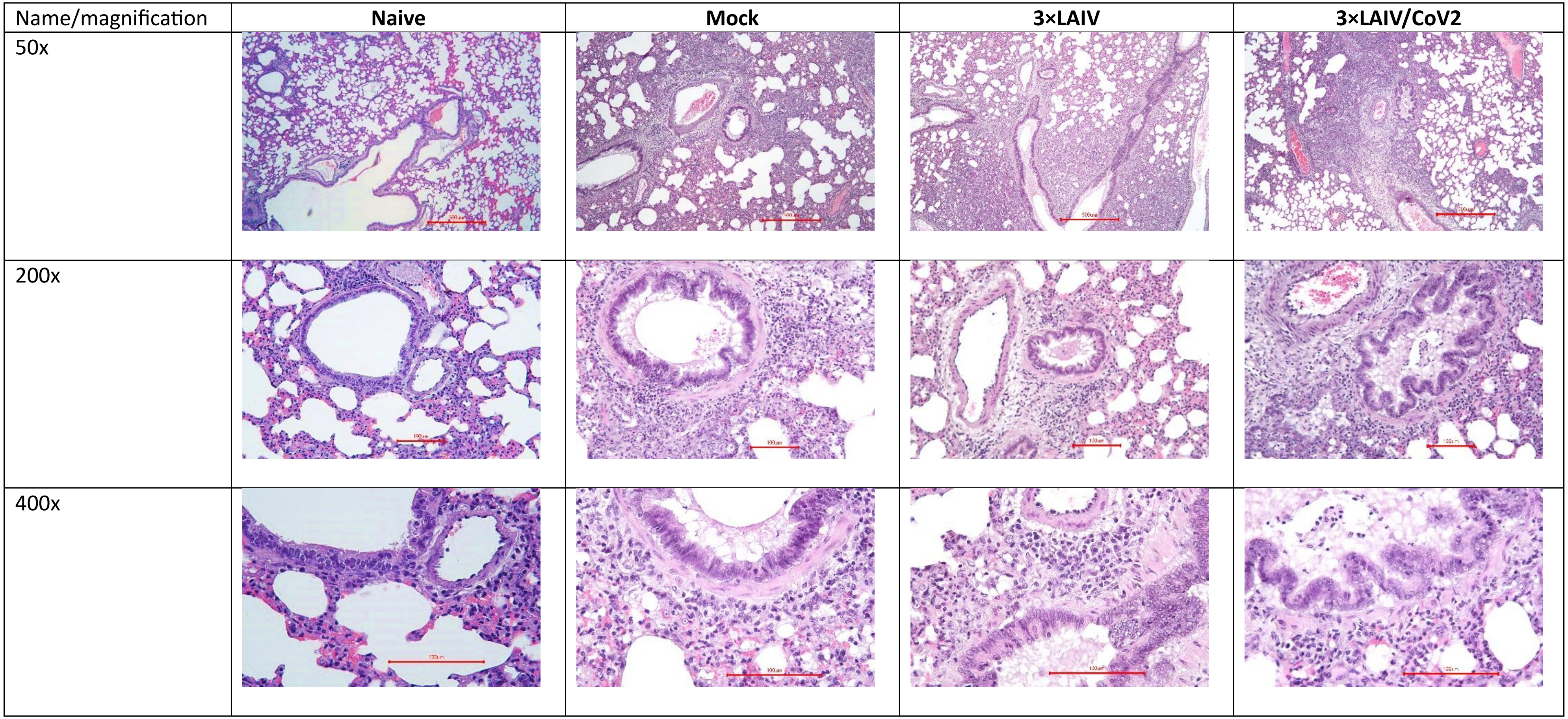

Figure S1. Histopathological assessment of protective effect of 3×LAIV/CoV2 against **Delta** challenge in a hamster model. Representative micrographs of hematoxylin-eosin-stained lung sections of animals on Day 6 after challenge are shown using magnification 50× (upper panel), 200× (middle panel) and 400× (lower panel).

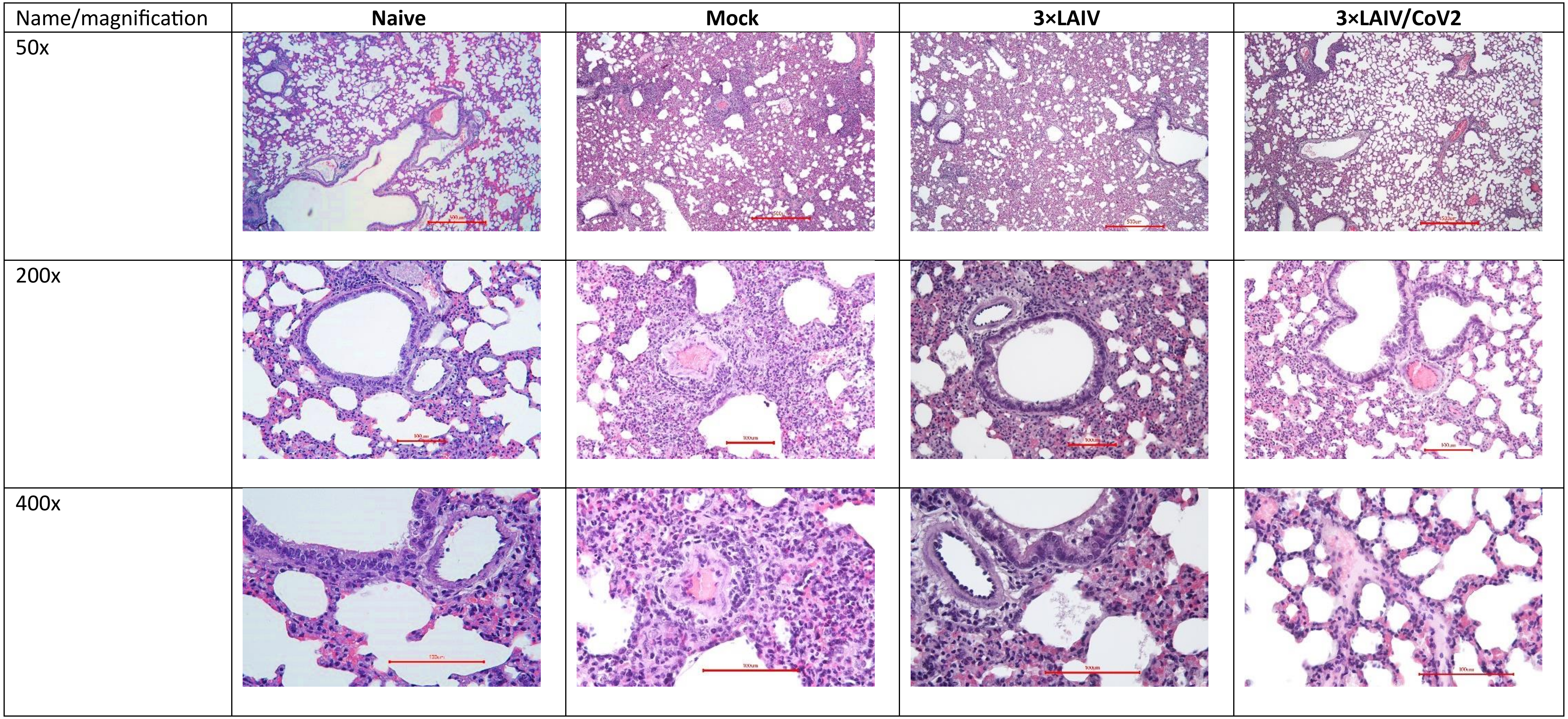

Figure S2. Histopathological assessment of protective effect of 3×LAIV/CoV2 against **Omicron** challenge in a hamster model. Representative micrographs of hematoxylin-eosin-stained lung sections of animals on Day 6 after challenge are shown using magnification 50× (upper panel), 200× (middle panel) and 400× (lower panel).

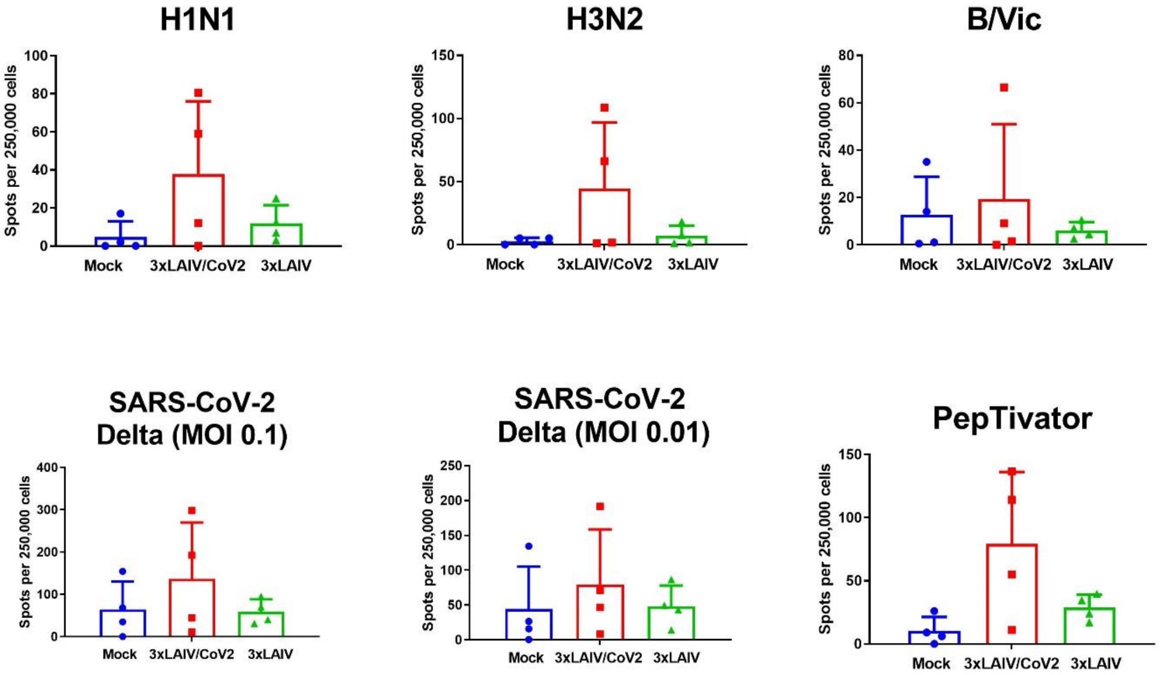

Figure S3. Levels of IFNγ-secreting cells in splenocytes of immunized Syrian hamsters on day 6 after infection with hCoV-19/Russia/SPE-RII-32759S/2021 (line B.1.617.2, Delta). Isolated splenocytes were stimulated in vitro with influenza viruses (upper panel), live SARS-CoV-2 or PepTivator (lower panel), followed by quantification of IFNγ-secreting cells using the Hamster IFN-γ ELISpot Plus kit.
